# Software tools for visualizing Hi-C data

**DOI:** 10.1186/s13059-017-1161-y

**Published:** 2017-02-03

**Authors:** Galip Gürkan Yardımcı, William Stafford Noble

**Affiliations:** 1 Department of Genome Sciences, University of Washington, 3720 15th Ave NE, Seattle, 98105 WA USA; 2 Department of Genome Sciences, Department of Computer Science and Engineering, University of Washington, 3720 15th Ave NE, Seattle, 98105 WA USA

## Abstract

High-throughput assays for measuring the three-dimensional (3D) configuration of DNA have provided unprecedented insights into the relationship between DNA 3D configuration and function. Data interpretation from assays such as ChIA-PET and Hi-C is challenging because the data is large and cannot be easily rendered using standard genome browsers. An effective Hi-C visualization tool must provide several visualization modes and be capable of viewing the data in conjunction with existing, complementary data. We review five software tools that do not require programming expertise. We summarize their complementary functionalities, and highlight which tool is best equipped for specific tasks.

**Electronic supplementary material:**

The online version of this article (doi:10.1186/s13059-017-1161-y) contains supplementary material, which is available to authorized users.

## Introduction

The three-dimensional (3D) conformation of the genome in the nucleus influences many key biological processes, such as transcriptional regulation and DNA replication timing. Over the past decade, chromosome conformation capture assays have been developed to characterize 3D contacts associated with a single locus (chromosome conformation capture (3C), chromosome conformation capture-on-chip (4C)) [1–3], a set of loci (chromosome conformation capture carbon copy (5C), chromatin interaction analysis by paired-end tag sequencing (ChIA-PET)) [4, 5] or the whole genome (Hi-C) [6]. Using these assays, researchers have profiled the conformation of chromatin in a variety of organisms and systems, which has revealed a hierarchical, domain-like organization of chromatin.

Here, we focus on the Hi-C assay and variants thereof, which provide a genome-wide view of chromosome conformation. The assay consists of five steps: (1) crosslinking DNA with formaldehyde, (2) cleaving cross-linked DNA with an endonuclease, (3) ligating the ends of cross-linked fragments to form a circular molecule marked with biotin, (4) shearing circular DNA and pulling down fragments marked with biotin, and (5) paired-end sequencing of the pulled-down fragments. A pair of sequence reads from a single ligated molecule map to two distinct regions of the genome, and the abundance of such fragments provides a measure of how frequently, within a population of cells, the two loci are in contact. Thus, by contrast with assays such as DNase-seq and chromatin immunoprecipitation sequencing (ChIP-seq) [7, 8], which yield a one-dimensional count vector across the genome, the output of Hi-C is a two-dimensional matrix of counts, with one entry for each pair of genomic loci. Production of this matrix involves a series of filtering and normalization steps (reviewed in [9] and [10]).

A critical parameter in Hi-C analysis pipelines is the effective resolution at which the data is analyzed [10, 11]. In this context, “resolution” simply refers to the size of the loci for which Hi-C counts are aggregated. At present, deep sequencing to achieve very high resolution data for large genomes is prohibitively expensive. A basepair resolution analysis of the human genome would require the aggregation of counts across a matrix of size approximately (3×10^9^)^2^=9×10^18^. Reads that fall within a contiguous genomic window are binned together, which reduces the size and sparsity of the matrix at the cost of resolution. Following this process, Hi-C data can be represented as a “contact matrix” *M*, where entry *M*_*ij*_ is the number of Hi-C read pairs, or contacts, between genomic locations designated by bin *i* and bin *j*.

Hi-C data presents substantial analytical challenges for researchers who study chromatin conformation. Filtering and normalization strategies can be employed to correct experimental artifacts and biases [9–11]. Statistical confidence measures can be estimated to identify sets of high confidence contacts [12]. Hi-C data can be compared with and correlated against complementary data sets measuring protein–DNA interactions, gene expression, and replication timing [13–15]. And 3D conformation of the DNA itself can be estimated from Hi-C data, with the potential to consider data derived from other assays or from multiple experimental conditions [16–19].

Efficient and accurate visualization of Hi-C data is not straightforward because Hi-C data is large and tools for the visualization of large-scale genomic data, such as genome browsers, do not directly generalize to visualizing data defined over pairs of loci [20, 21]. Furthermore, many biological hypotheses involve several biological processes and hence require the joint visualization of Hi-C data with other chromatin features. Thus, the visualization of Hi-C data alone is not sufficient—for a tool to be effective it must integrate different types of genomic data and annotations.

To address these challenges, a variety of software tools have been described recently that provide robust and informative methods for the interpretation of Hi-C data. Here, we investigate five tools that can be operated using a web browser or a graphical user interface: Hi-Browse v1.6 [22], my5C [23], Juicebox v1.5 [24], the Epigenome Browser v40.6 [25] and the 3D Genome Browser [26] (Table 1). These tools do not require programming expertise, and are more readily accessible. We assess these tools using several criteria, such as the types of visualizations provided by the tool, the ability to integrate many visualization modes, and the number and variety of datasets available in a given tool. In particular, we describe the suitability of each tool to different types of inquiry regarding the 3D structure of the genome and its interplay with other biological processes. We present examples that range from large scale visualizations of Hi-C data from whole genomes and chromosomes to fine scale local visualizations of putative promoter enhancer interactions and DNA loops, and highlight additional tool-specific capabilities that complement each visualization type.

**Table 1:** Comparison of toolkit functionality

	Hi-Browse	Juicebox	my5C	3D genome browser	Epigenome browser
Hi-C visualization					
Intrachromosomal heat map	$\checkmark $	$\checkmark $	$\checkmark $		
Interchromosomal heat map	$\checkmark $	$\checkmark $	$\checkmark $		
Circular plot	$\checkmark $				$\checkmark $
Rotated local heat map				$\checkmark $	$\checkmark $
Local arc track				$\checkmark $	$\checkmark $
Locus-specific circular plot					$\checkmark $
Virtual 4C plot		$\checkmark $	$\checkmark $	$\checkmark $	
Multi-dataset visualizations		$\checkmark $	$\checkmark $	$\checkmark $	$\checkmark $
Hi-C signal transformations	$\checkmark $	$\checkmark $	$\checkmark $		
Supplemental data visualization					
Supplemental data visualization		$\checkmark $		$\checkmark $	$\checkmark $
2D heat map features		$\checkmark $			
Continuous-valued tracks		$\checkmark $		$\checkmark $	$\checkmark $
Genome browser interface				$\checkmark $	$\checkmark $
Format for uploaded Hi-C data					
Sparse tab-delimited					$\checkmark $
Dense tab-delimited	$\checkmark $		$\checkmark $		
Sparse binary		$\checkmark $		$\checkmark $	$\checkmark $
Pre-loaded Hi-C data sets					
Rao et al. 2014		$\checkmark $		$\checkmark $	
Dixon et al. 2012	$\checkmark $	$\checkmark $		$\checkmark $	$\checkmark $
Lieberman-Aiden et al. 2009	$\checkmark $	$\checkmark $			$\checkmark $
Normalized versions	$\checkmark $	$\checkmark $			$\checkmark $
Supplemental data sets					
Annotations		$\checkmark $		$\checkmark $	$\checkmark $
ENCODE tracks		$\checkmark $		$\checkmark $	$\checkmark $
Roadmap Epigenome tracks					$\checkmark $
Implementation					
Free	$\checkmark $	$\checkmark $	$\checkmark $	$\checkmark $	$\checkmark $
Open source	$\checkmark $	$\checkmark $			$\checkmark $
Local installation option		$\checkmark $			$\checkmark $
Wiki					$\checkmark $
Browser interface	$\checkmark $		$\checkmark $	$\checkmark $	$\checkmark $
Java interface		$\checkmark $			

## Large scale visualization

The three-dimensional conformation of a complete chromosome or genome is usually visualized by one of two different methods. The contact matrix can be represented as a square heat map, where the color corresponds to the contact count, or the genome can be represented as a circle, with contacts indicated by edges connecting distal pairs of loci. Alternative large-scale visualizations are feasible, using for example a graph with nodes as loci and edges as contacts, but they have not proved as useful as heat maps and circular plots.

**Fig. 1:**
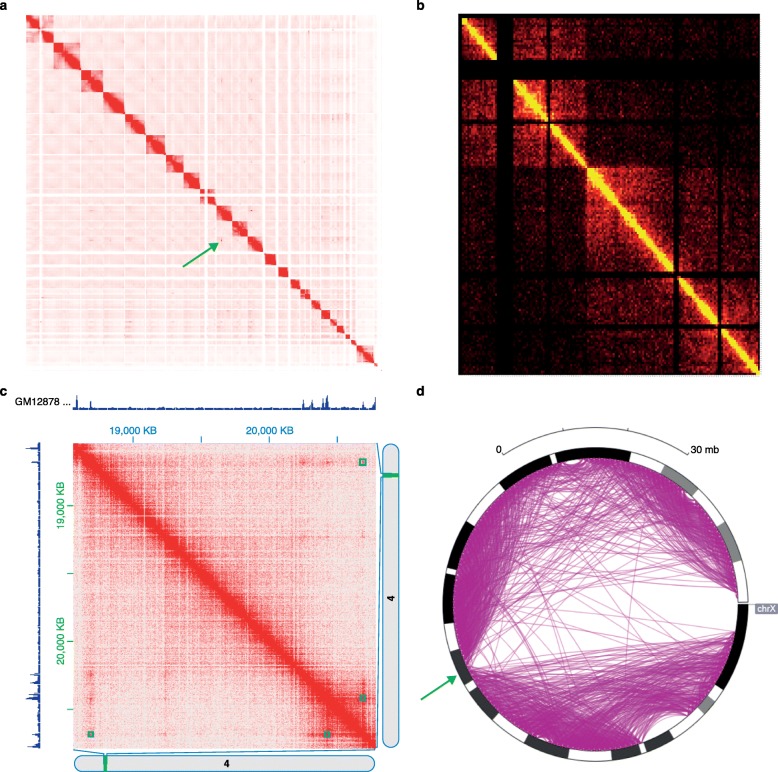
Heat map and circular plot visualization of Hi-C data. **a** Hi-C interactions among all chromosomes from G401 human kidney cells, as plotted by my5C. The *green arrow points* to aberrant interchromosomal signal in the Hi-C matrix, possibly caused by a rearragement event. **b** Heat map visualization illustrating the bipartite structure of the mouse X chromosome, as plotted by Hi-Browse, using in-situ DNase Hi-C data [49]. **c** Heat map visualization of a 3 Mbp locus (chr4:18000000-21000000) reveals the presence of loops that coincide with CTCF binding sites, validated by CTCF peaks shown on the top and left of the heat map. Computationally annotated loops are displayed as blue squares in the heat map. This heat map was produced by Juicebox, using in-situ Hi-C data from the GM12878 cell line [28]. **d** Circular plot of the bipartite mouse X chromosome, which shows a striking depletion of arcs between the two mega-domains, the locus that separates the mega-domains is shown by a green arrow. The plot was generated by the Epigenome Browser

A heat map is perhaps the most straightforward visualization method for a Hi-C contact matrix. Contact matrices are by definition symmetric around the diagonal, and the number of rows and columns is equal to the length of the genome divided by the bin size. The color scale associated with the heat map might correspond to raw contact counts or counts that have been appropriately normalized. The dominant visual feature in every Hi-C heat map is the strong diagonal, which represents the 3D proximity of pairs of loci that are adjacent in genomic coordinates. Heat maps can be constructed for the full genome (Fig. 1a) or for individual chromosomes (Fig. 1 b). Low resolution (1–10 Mb) contact matrices are typically sufficient for full genome visualizations and can be produced, for the human genome, using Hi-C datasets that contain tens of millions of read pairs. Whole genome visualizations can reveal potential rearrangements of the genome (Fig. 1 a), whereas single chromosome visualizations are useful for the identification of large-scale properties of chromatin conformation, such as chromosome compartments or the bipartite structure of the mouse inactive X chromosome (Fig. 1 b). Three of the five tools that we investigated—Hi-Browse, Juicebox, and my5C—provide heat map visualizations.

A heat map is also used to visualize the conformation of a locus of interest. The user can zoom into a region of the full contact matrix, visualized at higher resolution. The resulting map is used to identify loops, i.e., distal regions of DNA that exhibit unusually high contact counts relative to neighboring pairs of loci. Loop annotations detected by loop-finding algorithms can be displayed directly on a Hi-C contact map by Juicebox. Loop formation depends on DNA binding of the CTCF protein [27]; therefore, joint visualization of CTCF binding data from a ChIP-seq assay alongside Hi-C data is desirable for the interpretation of possible loops. Juicebox can plot data from other assays or genomic features, either as binary features or continuous signal plots, placing them on the sides of the heat map (Fig. 1 c).

Circular plots, originally designed to visualize genomic data, provide an alternative way to visualize Hi-C data on the chromosome scale. The circle typically represents the full length of a chromosome, and Hi-C contacts are represented by arcs (Fig. 1 d). The conversion of a contact matrix to a circular plot is straightforward: loci *i* and *j* are connected by an arc if entry *M*_*ij*_ in the contact matrix exceeds a user-specified cutoff value. Hi-Browse and the Epigenome Browser both generate circular plots.

## Local visualization

Hi-C data spans the full genome, however many hypotheses require the close inspection of a particular region or regions of interest. A common way to visualize several genomic data sets at a particular locus is via a genome browser, in which the DNA is arrayed horizontally and various types of data appear in parallel with the DNA sequence. The 3D Genome Browser and the Epigenome Browser extend the browser framework to incorporate Hi-C data, which provides rich and complex representations of DNA sequence, chromatin, gene structure, regulatory elements, and 3D conformation.

**Fig. 2:**
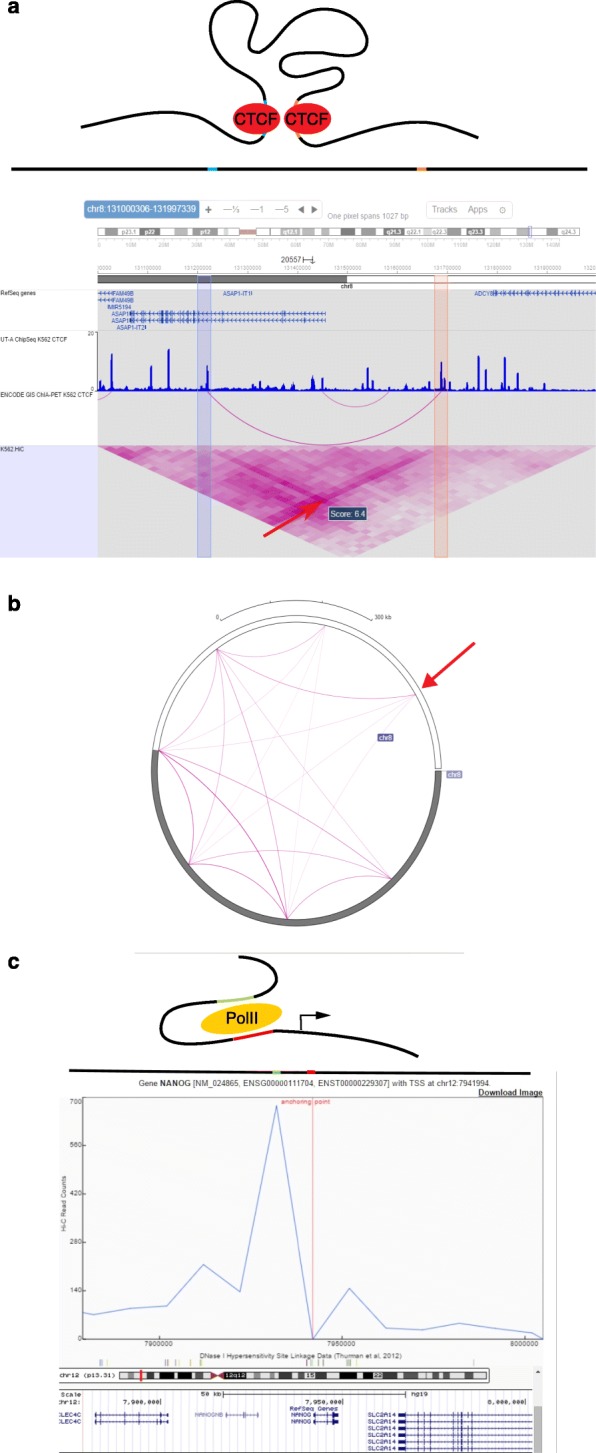
Local visualization modes. **a** A cartoon representation of the three-dimensional conformation of a putative DNA loop tethered by two CTCF proteins. CTCF binding sites are colored in blue and pink on the black DNA strand. Below the cartoon, a one-dimensional representation of the DNA fragment that forms the loop is placed above an Epigenome Browser visualization of a ∼1Mb locus, displaying the genes, CTCF binding, and interactions detected by ChIP-seq [50] and ChIA-PET assays (unpublished, GEO ID:GSM970216), and three-dimensional interactions as measured by Hi-C [28]. Two bins containing putative binding sites (*pink and blue bars*) show an enrichment of Hi-C contacts in the heat map visualization [28] (indicated by the *red arrow*). CTCF tethered interactions measured by ChIA-PET in an arcs view also indicate an interaction between these two putative binding sites. **b** A circular plot that shows the chromosome-wide long range contacts of the CTCF loop in panel a; the locus of interest is highlighted by a red arrow. The contacts are displayed as arcs, and only contacts above a certain threshold are visualized. **c** A putative promoter–enhancer interaction around the NANOG gene is displayed as a cartoon, which includes the PolII complex (*yellow oval*). *Red and green bars* in these cartoons represent the promoter and enhancer elements, respectively. Below the cartoon representations, a virtual 4C plot from the three-dimensional genome browser is shown, which visualizes the Hi-C signal around the NANOG promoter with a 1D representation of this region aligned above the plot. The bin in focus (the “anchoring point”) corresponds to the promoter of the NANOG gene. The height of the *blue line* indicates, for each locus, the read count for contacts between the current locus and the anchor point. In particular, the series shows an upstream enrichment of signal from a capture Hi-C experiment specifically targeting the NANOG promoter [51], which suggests a promoter–enhancer interaction. This observation is further supported by enrichment of DNaseI linkage data [41] (shown in grey below the primary plot) around the promoter and upstream regions. The NANOG gene is shown in the UCSC Genome Browser track under the virtual 4C plot

Four different visualization modes are available in the context of a genome browser. First, the heat map visualization, in which the upper triangle of the contact matrix is rotated by 45 degrees and then aligned so that the bins of the matrix correspond to chromosomal coordinates (Fig. 2 a). Both the 3D Genome Browser and the Epigenome Browser provide this visualization mode. However, heat map visualization is limited to capturing intra-chromosomal contacts, and the genomic distance between contacts is limited by the vertical screen space available to the heat map track. The display of distal contacts at high resolution is therefore impractical.

Second, the local arc track, similar to a circular plot, connects two genomic loci with an arc if the corresponding Hi-C signal is above a user-specified threshold (Fig. 2 a). Compared to heat map tracks, arc tracks offer a simpler interpretation of Hi-C contacts, at the expense of leaving out some of the data. The 3D Genome Browser and the Epigenome Browser also provide this visualization mode. The Epigenome Browser can display both Hi-C and ChIA-PET interactions in arc view, whereas the 3D Genome Browser uses arc tracks exclusively for ChIA-PET interactions.

Third, the global circular plot, which is intermediate between a local and global view includes contacts between a selected locus, (shown by a red arrow in Fig. 2 b) and the rest of the genome or a single chromosome. This plot provides a simpler way to visualize relevant long distance genome-wide contacts that involve a specific locus. The Epigenome Browser provides this visualization mode.

Fourth, the virtual 4C plot, is a slight modification of the local arc track (Fig. 2 c). Unlike a local arc track, which shows all contacts whose start and end loci are contained within the current browser view, a virtual 4C plot restricts the set of arcs to those that involve a single user-specified locus. Thus, a virtual 4C plot for the locus corresponding to bin *i* is equivalent to plotting the entries from the *i* ^*th*^ row of the contact matrix. By focusing on a single locus, a virtual 4C plot is used to test specific hypotheses regarding the bin of interest. The 3D Genome Browser provides this visualization mode. Juicebox and my5C offer a limited version of a 4C plot in the form of a track alongside a heat map visualization.

All four local visualization modes are particularly useful within the context of a full genome browser where, for example, potential regulatory contacts can be easily inspected alongside gene annotations, histone ChIP-seq experiments that mark enhancers and promoters, etc. For example, the Epigenome Browser can provide a view of a potential CTCF-tethered loop alongside multiple tracks: gene annotations, Hi-C and ChIA-PET contacts and CTCF ChIP-seq signal (Fig. 2 a). The resulting visualization plot is a concise and rich representation of multiple types of data, which strengthens the evidence for the existence of a DNA loop.

## Data availability

Input of data into a Hi-C visualization tool can be achieved in two ways: the data is pre-loaded by the tool developers or the user is responsible for uploading their own data. Both modes of data entry can be provided in a single tool. Here, we describe available data sets and upload capabilities for the five software tools, which includes both Hi-C data sets and auxiliary genomic data sets.

### Hi-C datasets

Four of the five visualization software tools come with publicly available datasets, but my5C does not. Available datasets include three influential studies that performed Hi-C experiments on several cell types, which we refer to using the last name of the first author on the respective publications: Lieberman-Aiden [6], Dixon [13], and Rao [28]. These three studies include nine human cell types from different lineages and tissues—IMR90, H1, GM06990, HMEC, NHEK, K562, HUVEC, HeLa, and KBM7—which makes them useful for many types of analyses. Datasets available for each tool are summarized in Table 1. Juicebox also offers datasets from 27 other studies, which includes data from a variety of organisms (Additional file 1). Most of these datasets are from Hi-C experiments performed on human cells, but each tool supports genomes of other organisms. The Epigenome Browser supports a total of 19 genomes, and the 3D Genome browser supports human and mouse genomes. The Hi-Browse, Juicebox, and my5C can be used with any genome.

Hi-C datasets are accumulating rapidly, and many users will need the capability to upload new datasets into these tools. All five visualization tools can upload user data or data downloaded from repositories such as 3DGD [29] or 4DGenome [30]. Most tools accept files that represent contact matrices; however, the file format requirements differ by tool (Table 1). The Epigenome Browser represents Hi-C matrices using tab-delimited text files, similar to the browser extensible data (BED) files often used in Genomics. Hi-Browse and my5C also uses tab delimited text files, but unlike the Epigenome Browser format, the my5C and Hi-Browse formats require that every entry be explicitly represented in the input file, which includes pairs of loci with zero contacts. The 3D Genome Browser uses its own sparse matrix representation in binary format, which can be created using the BUTLRTools software package [31]. Juicebox uses a complementary software package, Juicer [32], to build.hic files that store binary contact matrices at different resolutions. These.hic files are built from sequenced read pair files from a Hi-C experiment. The Epigenome Browser also supports the.hic format.

As Hi-C datasets continue to accumulate, the scientific community will likely come to a consensus on standardized file formats to represent Hi-C datasets. Most of the present file formats are very similar to one another, and conversion between most formats is straightforward using command line tools. An important tradeoff between different formats is the size of the file; sparse representations and especially the binary BUTLR and.hic formats require less disk space relative to uncompressed versions of other file formats.

### Data handling

Hi-C data sets can be binned at different resolutions. Generally, the user chooses a resolution value (i.e., bin size) based on sequencing depth of the dataset, striking a balance between detail and the sparsity that results from high resolution analysis. All tools in this review support visualization of Hi-C matrices at different resolutions. Datasets for each tool are stored at different resolution values, typically from 1 Mb to 5 kb. For user-uploaded datasets, the user is responsible for generating contact matrices at different resolutions, except for the.hic format which stores multiple resolutions in a single file.

After the resolution is set by the user, Hi-C data can be transformed to focus on different features of the data. The three most common transformations are matrix balancing to remove bin-specific biases [33–36], calculation of a correlation matrix for visualization of A and B compartments [6, 37], and calculation of the ratio of observed over expected Hi-C counts to account for the so-called “genomic distance effect” (the density of interactions close to the diagonal in the Hi-C matrix) [6]. Hi-Browse can transform raw Hi-C contact matrix into a (log) correlation matrix, whereas my5C generates the expected Hi-C signal and the ratio of observed to expected Hi-C signal. Juicebox indirectly performs all three transformations through the Juicer software. Other tools require the user to externally apply the transformations to the raw Hi-C data prior to upload.

Several software tools are available to carry out these external transformations. Juicer is the complementary software package to Juicebox that processes sequencing reads from a Hi-C experiment into.hic files that contain contact matrices at different resolutions and in various transformations. HiC-Pro [38] offers similar capabilities to Juicer but uses a tab-delimited sparse matrix format to store the output, which can be converted to.hic format. The HOMER suite of tools can generate dense Hi-C contact matrices and supports a rich set of downstream operations for transforming and analyzing Hi-C data [39]. Ay and Noble [9] provide a full review of Hi-C processing tools.

Certain tools visualize or compare multiple datasets simultaneously, a useful capability for investigating changes in 3D conformation of chromatin across different cell types or conditions. Juicebox and my5C can load two datasets, which allows the user to flip between heat map visualizations and visualizing the ratio of Hi-C signals in the two data sets. The 3D Genome Browser visualizes two Hi-C datasets as individual tracks. The Epigenome Browser offers the same capability for multiple datasets. Hi-Browse currently supports visualization of a single Hi-C dataset; however, Hi-Browse offers a method to identify statistically significant differential regions based on edgeR [40].

### Complementary datasets

The integration and visualization of different types of genomic data with Hi-C data is essential to interpret the interplay between biological processes such as chromatin conformation and gene regulation. Because the Epigenome Browser and the 3D Genome Browser specialize in this task, these tools provide many publicly available datasets, primarily generated by the ENCODE and Roadmap Epigenomics consortia. Furthermore, many relevant annotation tracks of various genomic features (genes, GC islands, repeat regions) are available, offering a rich collection of features that can assist in the interpretation of Hi-C data. Although Juicebox does not provide browser-like capabilities, the tool does offer a collection of genomic features, which allows a degree of joint visualization by placing tracks on the edges of the heat map visualization (Fig. 1 c). The my5C tool generates links to the UCSC Genome Browser for loci of interest, which allows the user to separately visualize other genomic features.

Tools that offer visualization of genomic features—Juicebox, the Epigenome Browser, and the 3D Genome Browser—also support the capability to upload user genomic data, such as gene annotations or ChIP-seq peaks. Well defined standards for file formats for such data types are already in place. These formats include the BED file format that defines genomic features relative to genomic intervals, and wig and bedgraph formats that are used to store continuous signal along the length of the genome.

As well as classic browser tracks, the 3D Genome Browser can visualize two other features that characterize 3D interactions: ChIA-PET and DNase-seq linkage annotations. ChIA-PET linkages are experimentally determined three dimensional contacts that are tethered by a specific protein [5], whereas DNase-seq linkages are predicted functional interactions between DNase hypersensitive sites [41]. These linkages are visualized as arcs and can aid in the interpretation of contacts revealed by a virtual 4C plot. For example, a virtual 4C plot focusing on the promoter of the NANOG gene displays a potential promoter–enhancer interaction upstream of the gene (Fig. 2 b).

## Implementation

All five tools differ fairly substantially in their functionality but also in how they are implemented. In particular, although all of the tools are freely available, only Hi-Browse, the Epigenome Browser, and Juicebox are open source. Furthermore, the Epigenome Browser and Juicebox can be installed to run on the user’s local computer, which circumvents the need to access online servers through the internet. This is desirable for analyses that require confidentiality or significant computational resources. Local installation for Juicebox requires only a 64-bit Java distribution, whereas installation of the Epigenome Browser depends on multiple software packages and server services, described in detailed, step-by-step instructions in the corresponding manual.

All of the tools provide a graphical user interface that is available through a web browser interface or via Java Web Start, and thus requires no or minimal installation. Unless a local installation is performed, all tools also require an internet connection. Access to tools that use a web browser interface is available through any operating system. For local installations, the Epigenome browser supports Linux and MacOS operating systems.

Documentation is provided for each of the five tools, although documentation of the 3D Genome Browser is being updated at present. The Epigenome Browser has its own wiki page that explains how to create and manage files for storing track information. Juicebox and the Epigenome browser have active online discussion groups that are maintained by the tool developers.

For each visualization tool, we profiled the speed of two important operations: loading user data and visualizing loci of sizes that are appropriate for both browser-based and heat-map-based tools (Table 2). Many factors, such as internet connection speed and server load, make it challenging to set up an exact benchmarking protocol; thus, we only report the approximate speed of loading operations, on the order of seconds, minutes or hours, and we report an average duration for visualization tasks. For benchmarking, we set the resolution parameter to either 40 kb or 50 kb, commonly used resolutions that strike a balance between sparsity and detail. We found that Juicebox, the Epigenome Browser and the 3D Genome Browser process user data in binary formats in a few seconds. Hi-Browse and my5C do not support loading of a complete dataset at these resolutions, instead the user must upload the Hi-C contact matrix corresponding to the region of interest. The average times required to visualize 1 Mb and 10 Mb heat maps showed that tools that do not use a browser framework are faster, with Juicebox and my5C the fastest tools. Browser-based tools are generally slower, especially for 10 Mb loci, consistent with the browser-based tools’ intended focus on local visualizations. We stress that user experience might differ from our benchmark due to differences in data sets, internet bandwidth and other parameters; thus, we offer this benchmark as a general guideline rather than an absolute measure of speed.

**Table 2:** Speed benchmarks for loading and visualizing Hi-C data

Tool	Loading	Visualization of	Visualization of		
	user data	1 Mb loci	10 Mb loci		
Juicebox	Seconds	1 s	1 s		
Hi-Browse	NA	10 s	86 s		
my5C	NA	1 s	3 s		
3D Genome Browser	Seconds	4 s	11 s		
Epigenome Browser	Seconds	33 s	73 s		

## Discussion

Each of the five tools discussed in this review aim to represent the same Hi-C data, but some tools are better suited to understanding the conformation of chromatin at large or small scales. Hi-Browse and my5C are well equipped to visualize large scale conformations, such as a complete genome or an individual chromosome. The Epigenome and 3D Genome browsers can better represent conformations at smaller scales, such as contacts that involve a single gene, which further enriches such visualization with other genomic features. Juicebox strikes a balance between these two approaches, and offers browser-like functionality to visualize supplemental data next to a matrix-based Hi-C visualization. Thus, the tool of choice for a Hi-C analysis task depends on the nature of the inquiry regarding chromatin conformation. In this review, we provide two example cases to illustrate our point: browsers are very capable of probing effects of chromatin conformation on the regulation of a single gene (Fig. 2), whereas heat maps are better suited to probing the overall organization of a single chromosome (Fig. 1).

All five tools offer a graphical user interface and do not require programming skills to operate, making them broadly accessible. However, although these tools are relatively straightforward to use to create sophisticated visualizations of Hi-C data, to process and convert Hi-C data into the required contact matrix format requires at least a basic understanding of programming. None of the visualization tools we reviewed offer the ability to process raw Hi-C reads into a contact matrix, but other toolkits are available to automate such tasks (reviewed in [9]). In addition to the tools we reviewed here, software packages such as HiCplotter [42] and HiTC [43] offer visualization capabilities but require programming capabilities.

We have discussed visualization of raw or normalized Hi-C data, but other transformations of the data can be visualized using the same set of tools. For example, statistical confidence measures, such as p-values produced by methods such as Fit-Hi-C [12] or diffHiC [44], can be converted to a contact matrix format and then visualized using the tools reviewed here. Hi-C data also can be used to infer the 3D structure of the chromatin (methods reviewed in [45]). The software tools reviewed here could be used to visualize the Euclidean distance matrix induced by such a 3D model. Direct visualization of the 3D models, especially in conjunction with other genomic features, is potentially very powerful. Several visualization tools for 3D genome structures are available, which include GMol [46], Shrec3D [18], TADBit [47] and TADKit [48].

## Additional file

Additional file 1 Contains a table listing the Hi-C experiments availble in Juicebox. (PDF 53 kb)

## Abbreviations

3C: Chromosome conformation capture
4C: Chromosome conformation capture-on-chip
5C: Chromosome conformation capture carbon copy
BED: Browser extensible data
ChIA-PET: Chromatin interaction analysis by paired-end tag sequencing
ChIP-seq: Chromatin immunoprecipitation sequencing
DNase-seq: Deoxyribonuclease I sequencing
CTCF: CCCTC-binding factor
ENCODE: Encyclopedia of DNA Elements
kb: Kilobase
Mb: Megabase
