# Supplementary material for: Software tools for visualizing Hi-C data
Source: Genome Biol. 2017 Feb 3;18:26. doi: 10.1186/s13059-017-1161-y (PMC5290626; doi:10.1186/s13059-017-1161-y)
Supplement: Additional file 1 — Contains a table listing the Hi-C experiments availble in Juicebox. (PDF 53 kb) [file 13059_2017_1161_MOESM1_ESM.pdf]

First author	Year	Journal	Cell types	Assay	Organism
Sanborn	2015	*PNAS*	HAP1	Capture Hi-C, COLA	Human
Darrow	2015	*PNAS*	RPE1, GM12878, AG08312, Patski	Hi-C	Human, Rhesus, Mouse
Kalhor	2011	*Nat Biotech*	GM12878	TCC	Human
Hou	2012	*Molecular Cell*	Kc167	Hi-C	Fruitfly
Li	2012	*Cell*	HCF116,HeLA,K562,MCF7,NB4	ChIA-PET	Human
Lin	2012	*Nature Immunology*	ProB	Hi-C	Mouse
Moissiard	2012	*Science*	Arabidopsis	Hi-C	Arabidopsis
Rickman	2012	*PNAS*	RWEP-1	Hi-C	Human
Sanyal	2012	*Nature*	GM12878	5-C	Human
Sexton	2012	*Cell*	Drosophila Embryo	Hi-C	Fruitly
Zhang	2012	*Cell*	ProB	Hi-C	Mouse
Jin	2012	*Nature*	IMR90	Hi-C	Human
McCord	2013	*Genome Research*	Fibroblast	Hi-C	Human
Nagano	2013	*Nature*	TH1	Single Cell Hi-C	Mouse
Naumova	2013	*Cell*	HFF,HelaS3,K562	Hi-C	Human
Seitan	2013	*Genome Research*	Thymocyte	Hi-C	Human
Sofuova	2013	*EMBO Journal*	neural stem cells, astrocytes	Hi-C	Mouse
Wang	2013	*PLoS One*	B-ALL,CALL-4,RL	Hi-C	Human
Zuin	2013	*PNAS*	HEK293T	Hi-C	Human
Ay	2014	*Genome Research*	3D7	Hi-C	*P. falciparium*
Feng	2014	*Molecular Cell*		Hi-C	Arabidopsis
Grob	2014	*Molecular Cell*		Hi-C	Arabidopsis
Ma	2014	*Nature Methods*	K562,H1	DNase Hi-C	Human
Rudan	2015	*Cell Reports*	Liver	Hi-C	Rabbit, Macaque, Mouse
Xie	2015	*Molecular Plant*	Arabidopsis	Hi-C	Arabidopsis

Table S1: **Chromosome conformation experiments available with Juicebox.**
